# Emotion regulation through bifocal processing of fear inducing and disgust inducing stimuli

**DOI:** 10.1186/s12868-020-00597-x

**Published:** 2020-11-23

**Authors:** Dina Wittfoth, Antonia Pfeiffer, Michael Bohne, Heinrich Lanfermann, Matthias Wittfoth

**Affiliations:** 1 Institut für Diagnostische und Interventionelle Neuroradiologie, Medizinische Hochschule Hannover, Hannover, Germany; 2 Fortbildungsinstitut für PEP, Tiedgestrasse 5, Hannover, Germany

**Keywords:** Emotion, Emotion regulation, Bifocal processing, Fear, Disgust, Functional magnetic resonance imaging

## Abstract

**Background:**

We present first-time evidence for the immediate neural and behavioral effects of bifocal emotional processing via visualized tapping for two different types of negative emotions (fear and disgust) in a sample of healthy participants.

**Results:**

Independent of stimulus type, neural activation in the amygdala is increased during regulation, while activation in the ventral anterior cingulate cortex is decreased. Behavioral responses, as well as lateral and medial occipital regions and the dorsolateral prefrontal cortex show differential regulatory effects with respect to stimulus type.

**Conclusions:**

Our findings suggest that emotion regulation through bifocal processing has a neural and behavioral signature that is distinct from previously investigated emotion regulation strategies. They support theoretical models of facilitated access to and processing of emotions during bifocal processing and suggest differential neural and behavioral effects for various types of negative emotions.

## Background

The ability to regulate emotions and other internal states is crucial for adaptive emotional functioning, and closely linked to subjective well-being [1–3]. Emotion regulation aims at influencing the type, intensity and duration of emotions using a variety of strategies [4]. The most common classifications found in the existing literature differentiate between explicit emotion regulatory strategies and implicit emotion regulatory strategies [4–6]. Other definitions differentiate between antecedent-focused emotion regulation and response-focused emotion regulation or between intrinsic (i.e. self-generated) emotion regulation and extrinsic (i.e. prompted by the environment) emotion regulation [4, 5, 7, 8].

Explicit emotion regulation, also called voluntary emotion regulation involves e.g. voluntary attentional control through selective attention or attentional deployment, cognitive change through reappraisal or detachment, behavioral suppression (keeping a ‘poker face’) or situation selection and modification [4, 5, 9, 10]. These strategies require conscious effort and monitoring, and usually involve some level of awareness and insight.

Implicit emotion regulation, sometimes also termed automatic emotion regulation is non-intentional, but has the goal of altering several or all aspects of an emotional response [7, 11–13]. Implicit emotion regulation is not conscious and does not involve deliberate control or monitoring. It is thought to be evoked by the stimulus itself [7, 11–13] and to be caused by a perceived discrepancy between the current emotional response and (unconscious) superordinate goals [8]. Implicit emotion regulation also involves a range of strategies such as automatic behavioral control (e.g. fear extinction or aversive conditioning), automatic attentional control such as performing a taxing cognitive task or automatic cognitive change such as repressive coping.

A substantial body of neuroimaging studies suggests that when emotion regulation is at play, a cortico-limbic network is characteristically involved. Most notably, amygdala activation is attenuated during regulation of negative emotions while the opposite pattern is observed in the medial and lateral ventral and/or dorsal prefrontal cortex, the anterior cingulate cortex (ACC), and inferior parietal regions. On the behavioral level, emotion regulatory strategies reduce the perceived negativity of unpleasant stimuli [4, 9, 10, 14].

Taken together, both the behavioral and the neural correlates of cognitive emotion regulation have been extensively studied [4, 5]. Aside from their cognitive, behavioral and motivational concomitants, emotions have long been recognized as full-body events [15–18]. However, considerably fewer studies investigate emotion regulatory strategies that directly involve the body. Neuroscientific research investigated the behavioral and neural correlates of expressive suppression, i.e. suppressing facial expressions in response to emotional stimulation (e.g. [7, 21, 22]). Two early reports by Gross and Levenson report that expressive suppression of negative emotions (i.e. keeping a ‘poker face’) does not change the subjective experience of sadness [20] and disgust [19]. A recent functional magnetic resonance imaging study investigating the effects of suppression and comparing it with other emotion regulation strategies found increased activation e.g. in the dorsolateral prefrontal cortex (dlPFC) which is commonly involved in explicit-controlled cognitive emotion regulation [10]. In the same study, expressive suppression also leads to decreased neural responses in the amygdala-hippocampal complex, and the temporo-occipital cortex subserving emotional perception and memory. A meta-analysis reviewing results from functional magnetic resonance imaging studies on emotion regulation subsumes expressive suppression under the term ‘body and response system’, i.e. response-focused regulation strategies [6]. The authors found that these types of strategies involve a network comprising e.g. the right temporo-parietal junction and the left vlPFC, both of which commonly subserve response-modulatory functions such as selective attention, reorienting or embodiment.

Above and beyond these studies, regulatory strategies that directly involve changes to the body (e.g. bifocal processing) remain somewhat understudied in the neuroscientific literature on emotion regulation in healthy populations [21], despite their effectiveness in clinical populations [22–30]. The relevance of the physiological components of emotional regulation is supported by results from studies investigating psychological disorders that are characterized by high levels of physiological arousal. Strategies involving direct manipulations of the body were effective in reducing symptoms of e.g. anxiety disorder or posttraumatic stress disorder (PTSD) [21, 26, 28]. Within the scope of body-centric emotion regulation strategies, bifocal (i.e. attention splitting) techniques involve simultaneous attention to negative emotional material and a concomitant, often alternating or rhythmic, physiological stimulation. They rely on an expansion of attention to include both negative emotional material and concomitant physiological stimulation at the same time (e.g. visual, auditory, and/or haptic stimulation). They also rely on highly standardized protocols, which lend themselves especially well to experimental investigation. Eye-movement desensitization and reprocessing (EMDR) for example uses rapid bilateral alternating stimulation, mostly in the visual domain, to reintegrate negative emotional experiences [27, 31]. EMDR influences emotional processing both on the behavioral level [22, 32] and on the neural level [21, 29]. Shapiro, who first developed EMDR, suggests a facilitation of access to and processing of negative emotional material which allows for the formation of new associations and reintegration of dissociated memories [27]. A recent functional magnetic resonance imaging study in healthy participants investigated how mono-aural and binaural alternating auditory stimulation influences the neural responses to and the subjective assessment of disgust inducing scenes [21]. Neural responses to negative stimuli with concomitant binaural alternating stimulation led to signal *in*creases in the amygdala, and signal *de*creases in the dorsolateral prefrontal cortex. Subjective measures of affectedness remained unchanged by both mono-aural and binaural stimulation. The findings by Herkt and colleagues [21] stand in contrast to the effects of cognitive emotion regulation, which commonly leads to increased prefrontal activation, decreased limbic activation and decreased ratings of subjective affectedness [4, 5]. They also support the notion that bifocal processing facilitates access to emotional processing, as mirrored by the increased limbic activation and decreased prefrontal activation.

Body tapping (i.e. the soft rhythmic palpitation of specific body points with one or more fingers) offers another bifocal approach for the regulation of strong emotional reactions which is widely used in various clinical settings [24, 26, 28, 33, 34]. Because of its simplicity, body tapping is also well suited for self-application in non-clinical settings [26, 35]. Additionally, interventions involving body tapping (e.g. Emotional Freedom Technique (EFT)) are effective in reducing symptoms of PTSD [22, 32, 36]. In a study in veterans with PTSD [37], only ten percent of the treatment group met the criteria for PTSD after six single EFT sessions, compared to 96 percent in the waitlist group. Only 14 percent of veterans in the waitlist group that still met the criteria for PTSD still did so after participating in six single EFT sessions. Additionally, EFT was shown to reduce sleep problems [37], pain [28], as well as symptoms of anxiety and depression [37]. These improvements remained stable in 3 months and 6 months follow-up [28, 37], and were replicated in two separate samples of veterans with PTSD [38] and subclinical PTSD [23].

Further study of bifocal approaches such as body tapping or EMDR for emotion regulation seems warranted given their relative simplicity and their robust effects in clinical populations detailed above. The results from Herkt and colleagues [21] furthermore suggest that a unique pattern of neural and behavioral responses may underlie the processing of aversive emotional stimuli during bifocal processing tasks, particularly in the initial phase of regulation. Investigations of the immediate and sustained neurophysiological effects and behavioral responses related to emotion regulation through bifocal processing are necessary to classify this technique with respect to other techniques of emotion regulation (compare [5]), and to develop models of its behavioral and clinical effectiveness [30].

### Study objective

The goal of this study is to investigate how bifocal processing influences neural processing and subjective ratings in response to negative emotions elicited by fear inducing and disgust inducing scenes. Based on previous findings from a study using an auditory EMDR task involving disgust inducing and neutral pictures [21] we hypothesize that emotion regulation through bifocal processing will entail greater limbic activation and decreased prefrontal activation compared to passive viewing of disgust inducing scenes. Moreover, we expand on previous results by introducing fear as an additional aversive condition. The regulation of fear is particularly relevant in the everyday life of healthy populations, and especially for the clinical application of bifocal processing strategies in clinical settings, e.g. when treating phobias, generalized anxiety disorder and PTSD [26, 30]. We also aim to investigate how the neural responses to bifocal processing through body tapping relate to existing models of the neural underpinnings of emotion regulation [4, 5, 13, 39].

## Materials and methods

### Participants

Twenty-one healthy participants took part in the study after providing written informed consent according to the Declaration of Helsinki. They were recruited from the student population of Hannover Medical School and Hannover University and received 20 Euro as compensation for their participation. Ethical approval was granted by Hannover Medical School’s Ethics Committee. Functional magnetic resonance imaging data, behavioral data, and questionnaire results from a subset of seventeen eligible participants (mean age = 23.47 years, standard deviation (SD) = 2.45, 8 female) were included in the final analysis. Four participants were excluded from the present analysis due to excessive movement (N = 3), or above cut-off depression scores (N = 1). On average, participants in our study sample scored low with respect to state anxiety (STAI-T [40]: mean = 32.00, SD = 6.29) and depressive symptoms (BDI-II [41]: mean = 2.53, SD = 2.77). Measures of habitual emotion regulation style were available for a subset of twelve subjects. Emotion Regulation Questionnaire (ERQ [42]) suppression and reappraisal scores were 3.35 (SD = .86) and 4.63 (SD = .74), respectively.

### Data acquisition and analysis

#### Study design and questionnaires

Participants attended two scanning sessions on the same day. Between the two scanning sessions, they were shown how to apply the body tapping sequence by a separate group of five lay instructors (mean age = 25.20 years, SD = 2.68, 2 female). The lay instructors were trained by either one of two skilled professionals (MB and MW) in applying the tapping sequence without further explanation regarding its purpose and effects. Instructions for body tapping were delivered to the study participants by one of the lay instructors based on a standardized script and included two consecutive rounds of tapping. Specifically, participants were instructed to ‘stay present with the picture while tapping’ and to be ‘curious to see what happens’. We informed participants that their experience might stay the same, change a little bit, or change a lot. We introduced tapping to the participants as an exercise rather than an emotion regulation tool. Neither the lay instructors nor the study participants were explicitly asked to regulate their emotions and were thus naïve to the purpose of tapping.

One tapping round consisted of the tapping of 16 body points, a short relaxation phase, and another sequence of tapping 16 body points [26, 35]. To collect subjective units of discomfort (SUD), we asked participants to rate the discomfort elicited by the image that they had perceived as the most negative image in the viewing condition. SUD were recorded on a scale from 0 (no discomfort) to 10 (strongest possible discomfort) at three different time points to assess changes in perceived negativity elicited by the respective picture pre-tapping, after one round of tapping and after two rounds of tapping. Twelve participants chose disgust inducing pictures as the most negative stimulus, while five participants perceived a fear inducing picture as the most negative stimulus.

The stimulus set consisted of 96 slides (32 fear, 32 disgust, and 32 neutral pictures) taken from the International Affective Picture System (IAPS [43]) as well as one of our previous studies (see Schardt et al. [9]). All stimuli were presented on an MR-compatible 40-inch screen (Nordic NeuroLab, Bergen, Norway) through Presentation^®^ Version 17.1 (Neurobehavioral Systems, Inc., Berkeley, CA, USA). We divided the stimuli into two sets of 48 pictures each and matched the sets for complexity, content, color and brightness within emotional conditions.

During the first run, participants were instructed to attentively view the first set of pictures and to let all upcoming emotions unfold naturally (viewing condition). We presented each stimulus in a slow event-related design for 8 s in pseudo-randomized order with no more than three consecutive trials from the same condition. Each picture was followed by a user-paced rating of negativity on an 8-point Likert scale from 0 (weak) to 7 (strong). Participants rated each picture’s negativity by pressing an MR-compatible response grip (Nordic NeuroLab, Bergen, Norway) with their left and right index fingers. A fixation cross was shown for four seconds after each rating. In the second functional imaging run, participants attentively viewed the second set of pictures while concomitantly visualizing tapping their three favorite body points (regulation condition). Again, they gave ratings of perceived negativity after each picture. The total duration of each functional imaging run was approximately 13 min.

Prior to the first functional magnetic resonance imaging (fMRI) session, participants filled in questionnaires regarding depressive symptoms (BDI-II), habitual emotion regulation strategies (ERQ), and trait anxiety (STAI-T). Before and after each scanning session, participants filled in questionnaires to assess changes in state anxiety (STAI-S [40]) related to the experimental procedure. We also assessed changes in state anxiety related to functional imaging. Mean STAI-S scores were compared in a repeated measures analysis of variance (ANOVA) with factors time (before scanning, after scanning) and session (viewing, tapping) at a threshold of p < .05. We calculated Bonferroni-corrected post hoc tests for significant main effects and interactions.

#### Behavioral data

For each subject we calculated mean in-scan negativity ratings for each experimental condition and mean tapping SUDs for each tapping round. We standardized mean scores by the number of possible answers minus one to ensure comparability between in-scan and SUD ratings.

Mean standardized in-scan negativity ratings were compared using a within-subject repeated-measures ANOVA with factors condition (viewing, tapping) and stimulus type (fear, disgust, neutral) at a significance level of p < .05. Mean standardized SUDs were compared by means of a Friedman-Test with the factor time (pre-tapping, post round 1, post round 2) at a significance level of p < .05. We employed Bonferroni-corrected post hoc Wilcoxon tests to assess for significant differences.

To assess the effectiveness of actual tapping versus visualized tapping, we compared measures of successful regulation during scanning with those recorded during the tapping instruction by calculating standardized mean difference scores. For in-scan negativity ratings, we computed Δfear = regulation fear–viewing fear, and Δdisgust = regulation disgust–viewing disgust. For SUDs, we calculated ΔSUD1 = post round one–pre-tapping, ΔSUD_2=_post round two–post round one, ΔSUD_tot_ = post round two–pre-tapping. We assessed the respective scores for significant differences by means of Bonferroni-corrected t-Tests for in-scan ratings, as well as Wilcoxon-Tests for SUDs and comparisons between Δfear versus ΔSUD_tot_ and Δdisgust versus ΔSUD_tot_.

#### Functional data

We obtained T2* weighted images using an echo-planar imaging (EPI) sequence with 38 slices and a voxel size of 2.3 × 2.3 × 3 mm and a distance factor of 10 percent (repetition time (TR) = 2.4 s, echo time (TE) = 30 ms, flip angle (FA) = 80°, field of view(FOV) = 240 mm). We recorded additional structural images using a Magnetization Prepared Rapid Gradient-Echo (MPRAGE) sequence with 192 slices and a voxel size of 1 × 1 x 1 mm and a distance factor of 50 percent (TR = 2.5 s, TE 4.37 ms, FA = 78°, FOV = 256 mm).

Functional brain imaging data were analyzed using Data Processing and Analysis for Brain Imaging (DPABI, [44]) and Statistical Parametric Mapping (SPM12) running on Matlab R2016b. Data were slice-timed to the second slice, realigned to the mean image, and coregistered to the high-resultion T1 image. We used DPABI’s internal quality check to exclude subjects with excessive motion artifacts. The T1 image was segmented, and the deformation fields of the segmentation were used to normalize the functional images and resample them to 2 mm^3^. In a subsequent preprocessing step, the functional images were smoothed using a Gaussian Kernel of 8 mm full width at half maximum (FWHM).

We compared differential effects of emotion regulation with respect to picture type in a second-level random effects group analysis within a 2 x 3 ANOVA with factors condition (viewing, regulation) and stimulus type (fear, disgust, neutral). To this end, we calculated main effects and the interaction effect of both factors, and additional t-contrasts as post hoc tests comparing fear inducing pictures, disgust inducing pictures and neutral pictures across conditions. Whole-brain results are reported at a family-wise error corrected intensity threshold of p < .05. We also investigated effects within the amygdala using a structural ROI derived from the AAL database implemented in the WFU PickAtlas 3.0.5b [45–47] at a family-wise error corrected threshold of p < .05. Since the FWE-corrected whole-brain analysis did not yield significant interactive effects in the dlPFC, we also report results from an exploratory analysis of the contrast comparing disgust regulation and neutral regulation (0 -1 1 0 1 -1) at a lower intensity threshold of p < .001 (uncorrected) with additional small volume corrections for multiple comparisons (FWE-corrected p < .05). Anatomical labels for cluster peaks are reported in Table 1 and Additional file 1: Table S1 along with the MNI coordinates, cluster sizes, and peak voxel statistics.

**Table 1:** Peak coordinates of clusters according to the Montreal Neurological Institute (MNI) stereotactic system and their respective Z-scores from the whole-brain analysis comparing fear-inducing, disgust-inducing and neutral pictures across regulation conditions at a family-wise error (FWE) corrected *p* < .05

		Effects of condition						Regulation > Viewing						Viewing > Regulation					
		Cluster size	p(FWE) voxel	Z	MNI-coordinates			Cluster size	p(FWE) voxel	Z	MNI-coordinates			Cluster size	p(FWE) voxel	Z	MNI-coordinates		
					x	y	z				x	y	z				x	y	z
Ventral Anterior Cingulate Cortex	28	1	0.039	4.89	− 6	28	0							3	0.020	5.02	− 6	28	0
	L	1	0.042	4.87	− 4	30	2												
*Region of Interest*																			
Amygdala	L	9	0.004	4.04	− 22	2	− 22	9	0.004	4.04	− 22	2	− 22						
	R	6	0.004	4.00	28	− 2	− 18	6	0.004	4.00	28	− 2	− 18						

		Effects of stimulus type						Fear > Disgust						Disgust > Fear					
		Cluster size	p(FWE) voxel	Z	MNI-coordinates			Cluster size	p(FWE) voxel	Z	MNI-coordinates			Cluster size	p(FWE) voxel	Z	MNI-coordinates		
					x	y	z				x	y	z				x	y	z
Inferior Frontal Gyrus, orbital part	L													31	0.000	6.01	− 24	36	− 10
Superior Frontal Gyrus, orbital part	R	11	0.004	5.39	22	30	− 14							25	0.001	5.73	22	30	− 14
Superior Frontal Gyrus	R	4	0.030	4.98	14	40	42												
Medial Superior Frontal Gyrus	R	26	0.007	5.28	14	48	26	7	0.015	5.09	14	48	28						
Precentral Gyrus	L	61	0.001	5.62	− 44	2	32												
	L	1	0.013	5.16	− 34	− 6	68												
	L	3	0.021	5.06	− 24	− 6	54	1	0.046	4.83	− 36	6	52						
Middle Frontal Gyrus	L	1	0.048	4.87	−38	18	52	1	0.048	4.82	-38	16	52						
	L	24	0.005	5.37	− 32	54	4	9	0.021	5.01	− 32	54	6						
	L	23	0.001	5.72	− 24	36	− 12												
Supramarginal Gyrus	L	462	0.000	7.53	− 66	− 22	34							319	0.000	7.21	− 66	− 22	34
	R	158	0.000	6.32	60	− 18	32							227	0.000	6.27	60	− 18	30
Inferior Temporal Gyrus	R	1	0.043	4.89	50	− 26	38												
	L	3900	0.000	Inf	−46	− 62	−6							131	0.000	6.72	− 46	− 60	− 8
	R	12	0.005	5.34	50	− 48	− 16												
Middle Temporal Gyrus	R	1510	0.000	7.69	50	− 62	10	1633	0.000	6.84	48	− 62	12						
	L							791	0.000	6.30	− 50	− 50	14						
	R	1	0.047	4.87	58	− 36	− 4	11	0.006	5.28	48	− 38	2						
Superior Parietal Lobule	R	85	0.000	5.82	26	− 58	50							37	0.005	5.32	26	− 56	48
	L	21	0.004	5.38	− 18	− 78	56												
Inferior Parietal Lobule	L	244	0.001	5.67	− 56	− 58	36												
	R	54	0.011	5.18	54	− 58	42												
Insula	R	4	0.026	5.00	40	− 2	0							32	0.003	5.44	40	− 4	2
	L													1	0.049	4.81	− 40	− 8	8
Amygdala	L	1	0.040	4.91	− 22	0	− 20												
Posterior Cingulate Cortex	R	3	0.032	4.96	4	− 50	30												
Posterior Mid-Cingulate Cortex	R	11	0.007	5.28	10	− 46	34	166	0.001	5.71	10	− 46	34						
		7	0.016	5.12	0	− 42	40												
Cuneus	R	5	0.039	4.91	6	− 88	20												
Lingual Gyrus	L													2101	0.000	7.63	− 22	− 72	− 12
Middle Occipital Gyrus	L	562	0.000	6.19	− 34	− 86	12							275	0.000	5.82	− 36	− 86	16
	R	17	0.014	5.13	30	− 78	22												
Cerebellum	L	3	0.042	4.90	− 38	32	16												
*Region of Interest*																			
Amygdala	L	49	0.000	4.91	− 22	0	− 20							36	0.001	4.39	− 20	0	− 20
	R	3	0.037	3.43	20	0	− 18							13	0.012	3.71	20	− 2	− 16

		Interaction condition x stimulus TYPE						Regulation: Fear vs. Neutral (− 1 0 1 1 0 − 1)						Regulation: Fear vs. Disgust (− 1 1 0 1 − 1 0)					
		Cluster size	p(FWE) voxel	Z	MNI-coordinates			Cluster size	p(FWE) voxel	Z	MNI-coordinates			Cluster size	p(FWE) voxel	Z	MNI-coordinates		
					x	y	z				x	y	z				x	y	z
Precuneus	R	124	0.000	6.17	20	− 54	16	145	0.000	6.24	20	− 54	16	53	0.001	5.67	10	− 52	14
Fusiform Gyrus	L	306	0.000	7.38	− 24	− 48	− 8	350	0.000	7.23	− 24	− 48	− 8	147	0.000	6.41	− 24	− 46	− 8
	R	267	0.000	7.75	26	− 46	− 6	281	0.000	7.32	26	− 48	− 6	168	0.000	7.31	28	− 46	− 6
	R	108	0.001	5.72	12	− 72	− 8												
	L							4	0.028	4.94	− 14	− 58	4						
Calcarine Cortex	L	183	0.000	6.66	− 6	− 86	0	533	0.000	6.96	− 6	− 86	0	5	0.016	5.07	− 6	− 88	− 2
	L	14	0.002	5.51	− 16	− 60	16	15	0.006	5.27	− 16	− 60	16	3	0.016	5.08	− 16	− 60	16
Middle Occipital Gyrus	L	561	0.000	Inf	− 34	− 82	28	747	0.000	Inf	− 34	− 82	28	35	0.003	5.41	− 34	− 82	28
	R	119	0.002	5.55	42	− 74	20	222	0.001	5.73	44	− 74	22						
Region of Interest																			

no suprathreshold voxels

## Results

### Effects of emotion regulation on behavioral measures of negativity and anxiety

In-scan negativity ratings showed differential regulation effects with respect to picture type (interaction condition x stimulus type F [2, 32] = 8.64; p = .001, eta^2^ = .351; see Additional file 1: Figure S1). Both fear inducing pictures (T [16] = 17.08, p < .001) and disgust inducing pictures (T [16] = 21.79, p < .001) were rated as significantly more unpleasant relative to neutral pictures. Fear inducing pictures and disgust inducing pictures were rated as equally negative in the viewing condition (p = .384). Negativity ratings were significantly decreased during regulation compared with viewing of disgust inducing pictures (T [16] = 6.67, p < .001), but only nominally decreased for fear inducing pictures (p = .089).

During the tapping instruction, SUD ratings showed a continuous reduction over time (chi^2^ = 15.43, df = 2, p < .001, N = 17; see Additional file 1: Figure S2). The decline was present post round one of tapping compared to pre-tapping (Z = −2.56, p = .011), and post round two of tapping compared with both post round one (Z = −2.67, p = .008) and pre-tapping (Z = −.281, p = .005). Regulatory effects did not differ between tapping rounds (Z = −.039, p = .969; round one mean = .039, SD = .049, round two mean = .035, SD = .055).

**Fig. 1:**
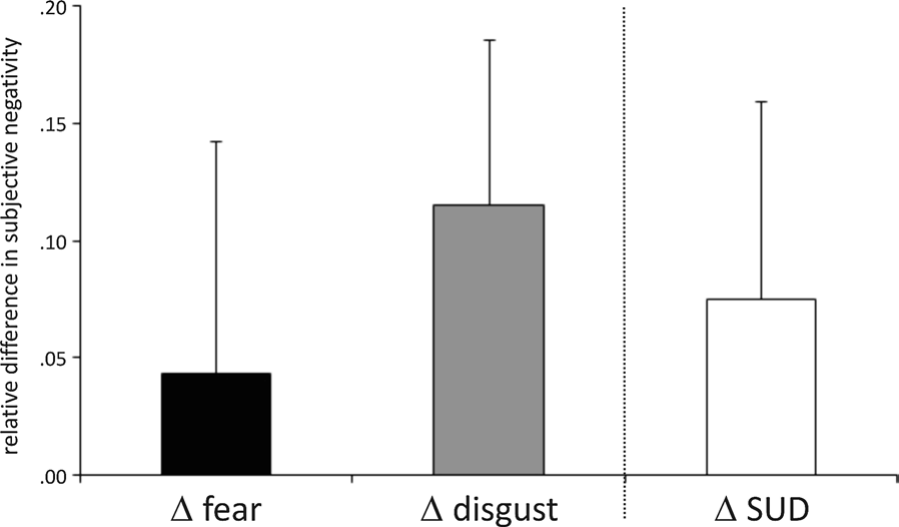
Regulatory effects of bifocal processing on subjective negativity. Left two bars: difference scores depicting the standardized mean difference between negativity ratings from the viewing condition minus the regulation condition for fear inducing pictures and for disgust inducing pictures during fMRI scanning. Rightmost bar: standardized mean difference between SUD ratings for the most aversive picture before tapping and after tapping. Greater regulation-related reductions in subjective negativity were observed for disgust inducing pictures versus fear inducing pictures. The effects of emotion regulation through visualized body tapping for both fear and disgust did not differ from regulation-related reductions in SUD during actual body tapping. SUD: subjective units of discomfort. fMRI: functional magnetic resonance imaging

Regulation-related reductions in negativity ratings for fear inducing pictures (Z = −1.042, p = .298; mean = .043, SD = .099) and disgust inducing pictures (Z = −1.444, p = .149; mean = .115, SD = .071) were comparable with the total reduction in SUDs after two rounds of tapping (mean = .075, SD = .085; Fig. 1). The regulation-related reduction in negativity ratings for fear inducing pictures was greater than the reduction observed for disgust inducing pictures (Z = −2.841, p = .005)..

Comparing before-and-after measures of state anxiety across the viewing and the regulation condition we found that STAI-S ratings differed with respect to both time point and run (interaction F [1, 16] = 9.946, p = .006, eta^2^ = .383; see Additional file 1: Figure S3). The pre-scan state anxiety measures did not differ between the viewing and the regulation condition (p = .332). State anxiety measures were increased after the viewing condition (T [16] = −4.709, p < .001), but remained unchanged after the regulation condition (p = .746). Participants reported significantly lower state anxiety measures after the regulation condition compared with the viewing condition (T [16] = 4.315, p = .001).

### Functional data

Anatomical labels, MNI coordinates, cluster sizes and statistical values for activated clusters from the FWE-corrected (p < .05) whole-brain analysis and from the amygdala ROI analysis (FWE-corrected p < .05) are summarized in Table 1. Additional information is available in the Additional file.

#### Emotion regulation

**Fig. 2:**
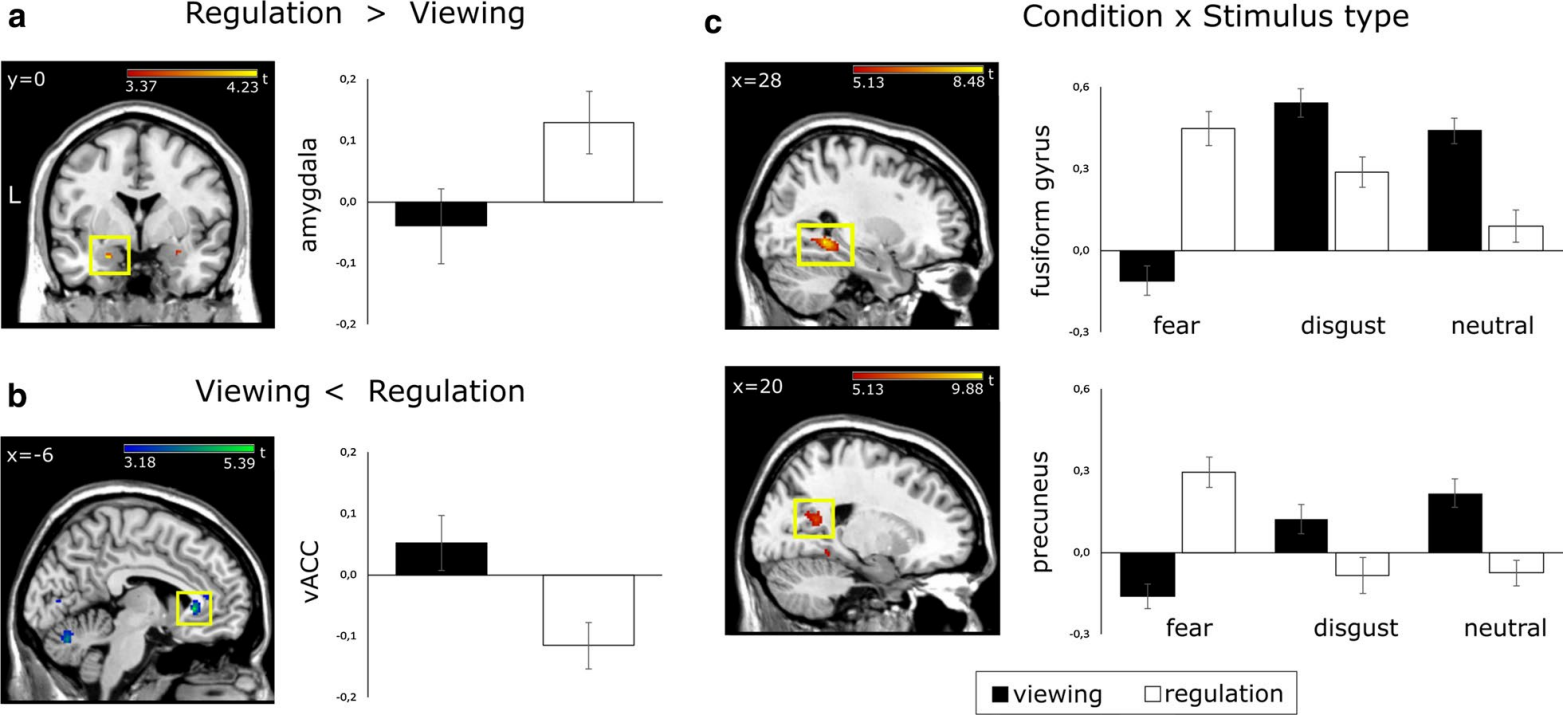
Neural effects of bifocal processing for fear inducing and disgust inducing emotional scenes. A regulation-related activation increase was found in the bilateral amygdala (**a**) while the vACC showed a regulation-related decrease in neural activation (**b**). The fusiform gyrus and the precuneus showed a regulation-related increase specifically in response to fear inducing stimuli (**c**). Amygdala ROI activation is displayed at a corrected p_FWE_ < .05. (**a**). Ventral anterior cingulate activation is shown at p < .001 uncorrected for display purposes (**b**). Activation from the whole-brain analysis is displayed at a corrected p_FWE_ < .05 (**c**). *FWE* family-wise error, *L* Left, *vACC* ventral anterior cingulate cortex; x left–right dimension in MNI space, y: front-back dimension in MNI space

We observed a main effect of condition in the amygdala and in the ventral anterior cingulate cortex (vACC). Activation during the regulation condition was greater relative to the viewing condition in the left and right amygdala (Fig. 2 and Table 1). The opposite pattern was found in the left vACC which showed greater activation during the viewing condition relative to the regulation condition (Fig. 2b and Table 1).

#### Emotion perception

The main effect of stimulus type revealed activation clusters in various regions underlying emotional processing, e.g. the amygdala, insula, lateral and medial prefrontal cortex, cingulate cortex, and temporo-parieto-occipital regions (compare Table 1).

Disgust inducing pictures were associated with greater activation in the orbital frontal cortex, the supramarginal gyrus, the lingual gyrus, the inferior temporal cortex, the superior and the inferior parietal lobule and the middle occipital cortex compared with fear inducing pictures. The bilateral amygdala and the bilateral insula also showed increased neural responses to disgust inducing pictures compared with fear inducing pictures. Conversely, the medial superior frontal gyrus, middle temporal gyrus and posterior middle cingulate cortex showed increased responses to fear inducing pictures compared with disgust inducing pictures.

#### Interaction of stimulus type and emotion regulation

We found that the effects of emotion regulation in the present paradigm differ with respect to stimulus type in the right precuneus, the bilateral fusiform gyrus, the left calcarine cortex, and the bilateral middle occipital gyrus.

In these regions, emotion regulation during fear inducing pictures led to an increase in neural activation. Emotion regulation during disgust inducing pictures and during neutral pictures on the other hand led to a decrease in neural activation (Fig. 2c).

Comparing the effects of emotion regulation on the processing of disgust inducing pictures versus neutral pictures, we found neural activation in several clusters in the dorsolateral prefrontal cortex. We also found clusters in the dorsal anterior cingulate cortex, the calcarine cortex, the middle occipital cortex and the cerebellum (exploratory analysis at an uncorrected intensity threshold of p < .001, all clusters surviving small volume correction for multiple comparisons (FWE-corrected p < .05); please see Additional file 1: Table S1 and Additional file 2: Figs. S4–S10 for additional information).

## Discussion

### Summary

The present study offers first-time evidence for the neural and behavioral correlates of visualized body tapping as a means of bifocal emotion regulation in response to negative emotional scenes in healthy participants. On the neural level, bifocal emotional processing (i.e. focusing both on the emotional material and the regulatory strategy) led to an overall increase of activation in the bilateral amygdala (Fig. 2a) and an overall decrease of activation in the ventral anterior cingulate cortex (Fig. 2b; compare also Table 1). Additionally, we found that emotion regulatory effects of bifocal processing were specific for fear in a set of lateral and medial temporo-parieto-occipital regions. Negativity ratings for fear inducing stimuli remained unchanged by emotion regulation, while negativity ratings were reduced for disgust inducing stimuli. Similar to previous work on bifocal emotional processing, we found that the dorsolateral prefrontal cortex also showed disgust-specific regulatory responses, albeit at a lower statistical threshold [21].

### Correlates of bifocal emotional processing

Our results support the view that bifocal emotional processing facilitates the re-integration of negative emotional material by increasing accessibility and re-organizing the underlying neural pathways [21, 26, 27, 48]. Increased activation of the amygdala during regulation suggests that emotional processing is preferentially performed while decreased activation of the ventral anterior cingulate cortex suggests that the automatic appraisal and accessing of mental representations usually triggered by emotional stimulation is attenuated [21, 49–51].

The vACC and more generally the ventromedial prefrontal cortex (vmPFC) appear to be antisympathetic and parasympathetic [52]. The vACC is involved in emotional conflict regulation and fear inhibition during extinction, as well as during reappraisal [50]. The vmPFC has been linked to diverse emotion regulatory functions, e.g. the maintenance of internalized representations of safety [49]. The vACC shows strong coupling with limbic and prefrontal areas [53] and its neural response pattern is anti-correlated with the response patterns of the amygdala, and with those of areas involved in cognitive and sensorimotor processing [50, 54, 55]. While the inverse relationship between regulation related responses in the amygdala and in the ventral anterior cingulate cortex due to body tapping requires further replication, it offers a possible explanation for the effectiveness of bifocal emotional processing in conditions characterized by high arousal such as general anxiety disorder or PTSD [22, 28, 32, 36]. In healthy participants, greater vmPFC activation and attenuated amygdala activation during reappraisal are associated with more normative declines in salivary cortisol in the home environment, suggesting that stress reponses are mediated by the interplay of these regions [56]. In PTSD and anxiety disorders, a dysregulation of the vACC has been observed e.g. in relation to fear conditioning, and the encoding of and reactivity to negative emotional material [57–59]. Indeed, the inverse relationship we observe between amygdala activation and vACC activation during bifocal emotional processing resembles the neural reponses to emotional-cognitive paradigms and symptom provocation found in patients with PTSD, and the neural responses to aversive stimuli found in patients with lesions in the vmPFC [60].

### The effects of stimulus type: fear versus disgust

As stated above, our results support the view that bifocal emotional processing facilitates access to and processing of negative emotional material, however particularly for fear inducing scenes (compare Fig. 2c and Table 1; [30]).

Participants in our study report the expected unaltered negativity ratings in response to fear regulation [21] and decreased negativity ratings for disgust regulation (Fig. 1). Fear perception was associated with greater activation in dorsal lateral and medial prefrontal regions underlying cognitive control and conflict monitoring [61, 62]. Fear regulation through bifocal processing specifically recruited basic and higher visual areas and regions subserving self-referential processes [55, 63, 64]. Disgust perception entailed neural responses in regions subserving bottom-up emotional processing and interoception, e.g. in the amygdala and in the insula [65, 66]. Disgust regulation on the other hand called on dorsolateral prefrontal regions underlying cognitive control and stimulus (re-)appraisal [67, 68].

The regulation related change in response to fear inducing scenes suggests a switch from top-down to bottom-upprocessing, which is also mirrored by the unchanged negativity ratings during fear regulation. The reduction in negativity ratings and the involvement of the dlPFC suggests the (uninstructed) recruitment of reappraisal processes during the regulation of disgust stimuli [49, 68]. Given the tentative nature of our analysis in the dorsolateral prefrontal cortex, these findings warrant cautious interpretation. More studies are needed to elucidate potentially disgust-specific regulatory effects in the dorsolateral prefrontal cortex in response to bifocal emotional processing.

In the absence of a specific regulatory strategy, emotion regulation may be evoked automatically by the stimuli themselves [12]. The emotion specific regulatory activation in the current paradigm might be mediated by the dimensions of picture valence (i.e. cognitive appraisal) and arousal (i.e. physiological/psychological affectedness) [62], possibly mirrored by the greater prefrontal activation in response to fear inducing scenes, and the greater limbic activation in response to disgust inducing scenes in the viewing condition. Since we did not specifically ask participants to distinguish between the dimensions of valence and arousal in their negativity ratings, our design does not permit direct investigation of this hypothesis. Future research should aim at separately addressing both valence and arousal in response to bifocal processing of different types of aversive emotional material. Investigating peripheral-physiological measures of arousal such as heart rate or skin conductance response might also be useful to elucidate the effects of bifocal emotional processing on the perception of arousal in response to different types of negative stimuli.

#### Bifocal emotional processing in the light of existing neuroscientific findings on emotion regulation

The behavioral and neural effects we report are unlikely to be related to habituation. If participants did habituate, we would expect to observe attenuated limbic responses, increased ventral and/or lateral prefrontal activation and decreased ratings of negativity (e.g. [57, 58]). However, in response to visualized tapping, we find sustained ratings of negativity for fear inducing stimuli, and unchanged self-reported anxiety scores. We also find activation increases in the limbic system subserving emotion perception [16, 69] and activation decreases in the ventral anterior cingulate cortex subserving e.g. reappraisal [50], antisympathetic/parasympathetic regulation [52] and fear conditioning [57].

Bifocal emotional processing involves manipulations of attention as does e.g. distraction, and manipulations of the body as does expressive suppression (compare 6). Both of these strategies consistently lead to decreased limbic (e.g. amygdala) activation, increased lateral prefrontal activation, and decreased measures of subjective affectedness [5, 10, 70, 71]. However, the neural and behavioral response patterns found in the present work differ from those cited above. Specifically, we did not observe an attenuation of amygdala activation and concomitant activation increase in lateral prefrontal areas that would be characteristic of emotion regulation through attentional deployment or suppression [6, 10, 70]. This might be related to the fact that our participants were neither using tapping as a means to distract themselves from the emotional material nor trying to control their bodily reactions to them. Rather, they were instructed to focus on both the emotional scenes and on tapping [26, 35]. Thus, the focus of attention was broadened, however not to the detriment of perceiving the emotion-evoking stimuli. The up-regulation of amygdala activation in the absence of lateral prefrontal activation suggests that participants did not actively attempt to increase their emotional responses [56]. However, keeping an internal focus (i.e. counting heartbeats) compared with an external focus (counting clicks) was previously shown to up-regulate amygdala activity without prefrontal involvement in anxiety-sensitive female participants [72].

Emotion-independent involvement of ventromedial prefrontal areas (rather than lateral prefrontal areas) suggests that neural responses to bifocal emotional processing resemble those to uninstructed emotion regulation [49]. Indeed, the present paradigm can be conceptualized to represent an incidental emotion regulation strategy since participants did not have the explicit goal to regulate emotions, and thus the effect of altering emotional responses was incidental [7, 12, 73]. However, the emotion-specific recruitment of the dorsolateral prefrontal cortex suggests that there might be differences in the amount of controlled regulation initiated by the bifocal processing of different types of emotional stimulation [12, 21].

Taken together, bifocal approaches seem to modulate both subjective appraisals and neural processing in response to negative emotional material [21]. However, the initial neural and behavioral responses seem to rely on overlapping but distinct psychological and neural mechanisms compared with previously investigated emotion regulation strategies [5, 6, 39, 73]. Possibly, bifocal emotional processing constitutes an automatic emotion regulatory process that incrementally introduces changes in affective responding that compound over time [5]. Future work should aim to identify exactly when and how the reduction in subjective experiences of negativity usually observed when applying bifocal strategies like EMDR or body tapping is brought about [26, 31].

## Limitations

The present study offers the first functional imaging investigation of the effects of visualized body tapping as an emotion regulation strategy in a healthy, young sample. Our work allows important, but limited inferences about the efficacy and the mechanisms of body tapping. Firstly, we used visualized rather than actual body tapping inside the fMRI scanner to limit motion artifacts due to tapping. This approach relied on neuroimaging studies that underscore the similarity between motor imagery and actual movement or touch [74–76]. However, since we did not directly measure brain activation during actual body tapping, we cannot rule out the possibility that actual body tapping might lead to different results compared with visualized body tapping. Our participants also visualized tapping their three favorite tapping points, which naturally varied across our sample and are thus heterogeneous. The behavioral results of our study nevertheless suggest that the actual location of body tapping is of secondary importance as both the visualization of tapping one’s three favorite tapping points and the physical performance of three complete tapping rounds with all 16 tapping points led to comparable reductions in subjective affectedness (Fig. 1). Further research should aim to elucidate the effects of actual compared with visualized tapping, and of different tapping locations on the behavioral and neural outcome of emotion regulation. Given the exploratory nature of this work, we investigated only healthy participants in a relatively young sample of university students (mean age of 25 years). We did this to facilitate the conceptualization of our results in the framework of preexisting neuroscientific findings of emotional regulation in healthy participants [4, 5, 7, 13, 39, 70]. Since our sample size is small and our participants are rather young, and since we did not include a clinical comparison group, the statistical power as well as the basis for extrapolations to general or clinical populations based on our results are limited.

## Conclusion

Taken together, our findings suggest that the visualization of body tapping as a means of bifocal emotional regulation is effective in altering the immediate neural and behavioral responses to negative emotional stimulation. Amygdala activation is increased during bifocal processing of emotional scenes, while activation in the ventral anterior cingulate cortex is decreased. However, subjective measures of negativity remain unchanged when regulating fear, while they are reduced when regulating disgust. We find modulation of dlPFC activation only for disgust, but not for fear. These findings stand in contrast to the increased prefrontal activation and the reduction in both amygdala activation and subjectively perceived negativity commonly observed during explicit- and implicit-controlled emotion regulation [4, 5, 7, 13]. However, our results fit in well with previous findings of increased amygdala activation, decreased dlPFC activation and unchanged subjective measures of negativity during bifocal processing of disgust inducing scenes [21].

To conclude, our results support the notion of increased accessibility, as well as re-integration of negative emotional material during bifocal processing [21, 26, 30, 31]. They also point to the utility of visualized body tapping in settings where movement is restricted or compromised, such as during functional imaging, dental exams, or in patients with movement disorders [74–76]. Our findings highlight the need for investigating bifocal processing approaches in various contexts and in larger samples. Future research should aim to elucidate neural and behavioral responses for different qualities of negative emotions (e.g. anger or sadness), active (e.g. body tapping) vs. passive (e.g. EMDR) administration of the secondary stimulation, effects in clinical populations, as well as the short- and long-term effects of emotional reintegration.

## Supplementary information

**Additional file 1: Table S1.** Results from whole-brain analysis at an FWE-corrected p<.05: Fear > Neutral, Disgust > Neutral, and from exploratory whole-brain analysis at p<.001 uncorrected, k=14: Regulation: Disgust vs. Neutral (0 -1 1 0 1 -1).

**Additional file 2: Figure S1.** Standardized mean in-scan negativity ratings; **Figure S2.** Standardized mean tapping SUD ratings; **Figure S3.** STAI-S before and after each fMRI session; **Figure S4.** Glass brain and plot of parameter estimates from the left Middle Frontal Gyrus (BA 48/46); **Figure S5.** Glass brain and plot of parameter estimates from the right Middle Frontal Gyrus (BA 46).

## Data Availability

The datasets used and/or analyzed during the current study are available in the Mendeley Data repository (10.17632/cm9xkbc8xp.2).

## Abbreviations

ACC: Anterior cingulate cortex
ANOVA: Analysis of variance
BDI-II: Beck’s depression inventory–version II
dlPFC: Dorsolateral prefrontal cortex
DPABI: Data processing and analysis for brain imaging
EMDR: Eye movement desensitization and reprocessing
EPI: Echo-planar imaging
ERQ: Emotion regulation questionnaire
FWE: Family-wise error
fMRI: Functional magnetic resonance imaging
FOV: Field of view
FWHM: Full width at half maximum
IAPS: International affective picture system
MNI: Montreal neurological institute
MPRAGE: Magnetization prepared rapid gradient-echo
PTSD: Post-traumatic stress disorder
SD: Standard deviation
SPM: Statistical parametric mapping
STAI-S: State trait anxiety inventory–state version
STAI-T: State trait anxiety inventory–trait version
SUD: Subjective units of discomfort
TE: Echo time
TR: Repetition time
vACC: Ventral anterior cingulate cortex
vmPFC: Ventromedial prefrontal cortex
