# Supplementary material for: Emotion regulation through bifocal processing of fear inducing and disgust inducing stimuli
Source: BMC Neurosci. 2020 Nov 23;21:47. doi: 10.1186/s12868-020-00597-x (PMC7681990; doi:10.1186/s12868-020-00597-x)

**ADDITIONAL FIGURES**

**In-scan ratings, SUD and questionnaire data**

Additional Figure 1: Standardized mean (±SD) in-scan negativity ratings

Additional Figure 2: Standardized mean (±SD) tapping SUD ratings

Additional Figure 3: STAI-S mean (±SD) scores before and after each fMRI Session

Additional Figure 4: Glass brain and plot of parameter estimates from the left Middle Frontal Gyrus (BA 48/46)


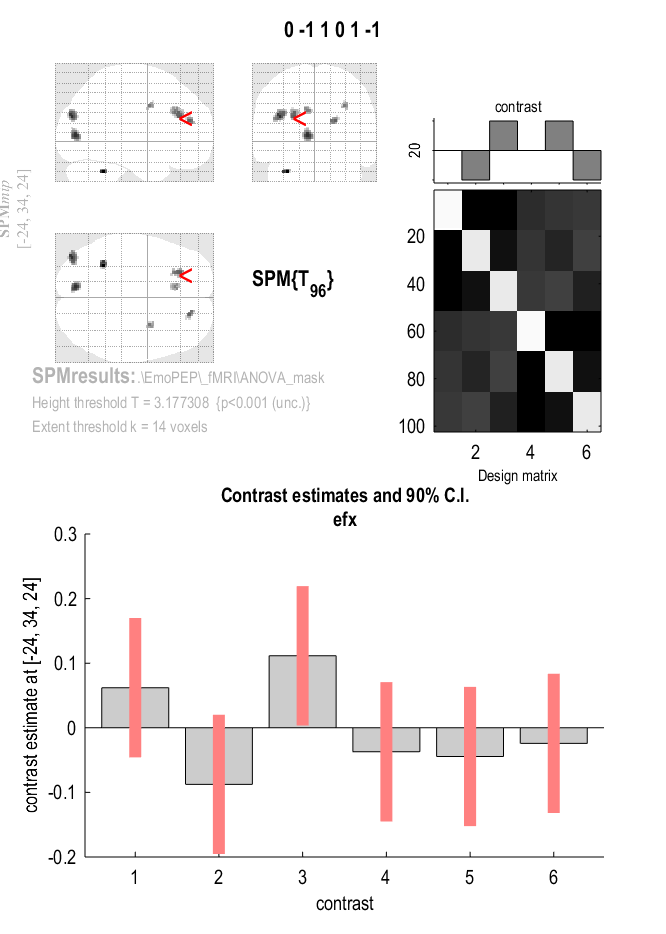


Additional Figure 5: Glass brain and plot of parameter estimates from the right Middle Frontal Gyrus (BA 46)


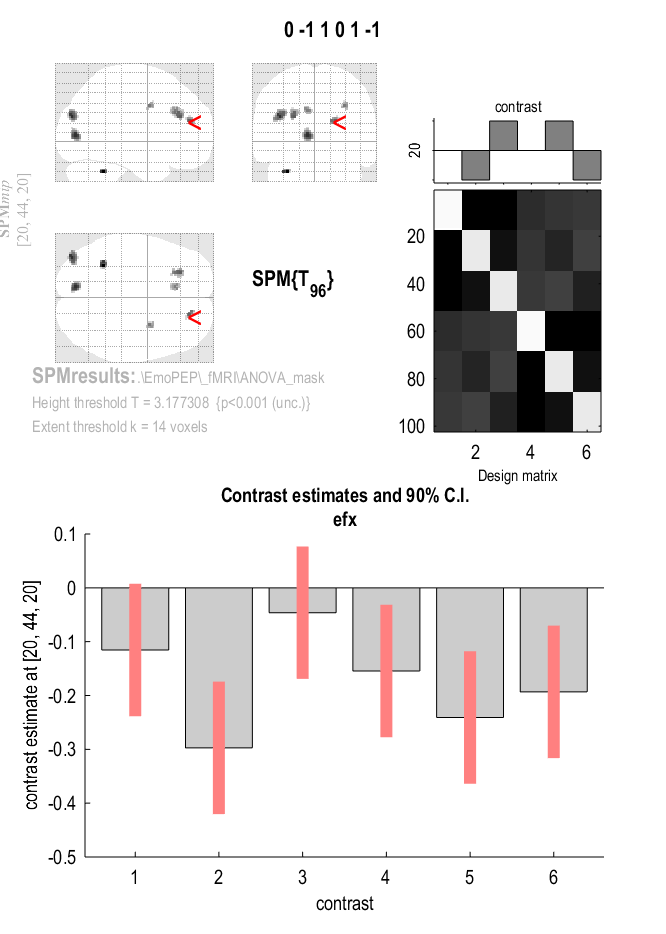

Supplement: Supplementary file 2 — Additional file 2: Figure S1. Standardized mean in-scan negativity ratings; Figure S2. Standardized mean tapping SUD ratings; Figure S3. STAI-S before and after each fMRI session; Figure S4. Glass brain and plot of parameter estimates from the left Middle Frontal Gyrus (BA 48/46); Figure S5. Glass brain and plot of parameter estimates from the right Middle Frontal Gyrus (BA 46). [file 12868_2020_597_MOESM2_ESM.docx]
